# Mothers’ quality of life delivering kangaroo mother care at Malawian hospitals: a qualitative study

**DOI:** 10.1186/s12955-021-01823-8

**Published:** 2021-07-28

**Authors:** Alinane Linda Nyondo-Mipando, Mai-Lei Woo Kinshella, Tamanda Hiwa, Sangwani Salimu, Mwai Banda, Marianne Vidler, Elizabeth Molyneux, Queen Dube, David M. Goldfarb, Kondwani Kawaza

**Affiliations:** 1 Department of Health Systems and Policy, School of Public Health and Family Medicine, College of Medicine, University of Malawi, Blantyre, Malawi; 2 Department of Obstetrics and Gynaecology, BC Children’s and Women’s Hospital,, University of British Columbia, Vancouver, Canada; 3 Department of Pediatrics and Child Health, College of Medicine, University of Malawi, Blantyre, Malawi; 4 Queen Elizabeth Central Hospital, Pediatrics, Blantyre, Malawi; 5 Department of Pathology and Laboratory Medicine, BC Children’s and Women’s Hospital, University of British Columbia, Vancouver, Canada

**Keywords:** Kangaroo mother care, Quality of life, Mothers, Malawi, Qualitative research

## Abstract

**Introduction:**

Kangaroo mother care is known to help save the lives of preterm and low birthweight infants, particularly in resource-limited health settings, yet barriers to implementation have been documented. Mothers and their families are very involved in the process of providing kangaroo mother care and the impact on their well-being has not been well explored. The objective of this research was to investigate the perspectives and experiences of a mother’s quality of life while delivering facility-based kangaroo mother care.

**Methods:**

This study is a secondary analysis of the qualitative data collected within the “Integrating a neonatal healthcare package for Malawi” project. Twenty-seven health workers and 24 caregivers engaged with kangaroo mother care at four hospitals in southern Malawi were interviewed between May–August 2019. All interviews were face-to-face and followed a topic guide. Content analysis was conducted on NVivo 12 (QSR International, Melbourne, Australia) based on the six World Health Organization Quality of Life domains (physical, psychological, level of independence, social relationships, environment, spirituality).

**Results:**

Fifty-one interviews were conducted with 24 caregivers and 14 health workers. Mothers experienced multidimensional challenges to their quality of life while delivering facility-based KMC. Though kangaroo mother care was considered a simple intervention, participants highlighted that continuous kangaroo mother care was difficult to practice. Kangaroo mother care was an exhausting experience for mothers due to being in one position for prolonged periods, compromised sleep, restricted movement, boredom, and isolation during their stay at the hospital as well as poor support for daily living needs such as food.

**Discussion:**

A heavy burden is placed on mothers who become the key person responsible for care during kangaroo mother care, especially in resource-limited health settings. More focus is needed on supporting caregivers during the delivery of kangaroo mother care through staff support, family inclusion, and conducive infrastructure.

**Supplementary Information:**

The online version contains supplementary material available at 10.1186/s12955-021-01823-8.

## Introduction

Kangaroo mother care (KMC) is an intervention that can help save the lives of preterm and low birthweight (LBW) infants [1]. At a rate of 12.0%, sub-Saharan Africa has the highest rate of preterm birth in the world, which has a global average of 10.6% [2]. KMC is a relatively low-cost and low-technology intervention compared to many other therapies for small and sick newborns in intensive or high dependency units [3, 4]. While it is accepted as a beneficial practice, implementation has faced multiple barriers [5–7]. Several challenges have been reported in the scale-up of KMC in sub-Saharan Africa, where there is an urgent need for KMC due to high rates of preterm birth and resource-limited health settings [8]. Caregivers are highly involved in the process of care [9–11], and recent research on facility-based KMC in Malawian hospitals revealed a reliance on mothers and their support networks, who take on responsibilities in care during hospitalization [12, 13]. Caregiver needs, such as food, bedding, and wraps to facilitate KMC, were not regularly provided by the hospital, and caregivers may face unintended consequences of admission due to responsibilities around the home and disrupt economic activities [12, 13].

While mothers are acknowledged to play a crucial role in the delivery of KMC and that poor support for caregivers is recognized as a major barrier to sustainable KMC practice, little is known about the personal toll on mothers. KMC was first developed in the early 1980s, yet there is still a gap in understanding the impact of facility-based KMC on mothers’ quality of life (QoL) and well-being over three decades later. Following the World Health Organization’s definition, QoL for a mother providing KMC may be conceptualized as perceptions of her position in life in relation to her goals, expectations, standards, and concerns as it relates to her culture, value systems, and context [14, 15]. Our recent review on KMC reported that several studies found that the discomfort associated with being in one position, the fear of how to handle a small baby, and the cultural inappropriateness associated with carrying a baby in front were some factors that affected the implementation of KMC [8]. An earlier review also reported similar findings that the QoL while implementing KMC was affected because of the required long hours that a mother had to give in even though the baby seemed to be comfortable [6]. The objective of this study is to investigate the perspectives and experiences of a mother’s QoL while delivering facility-based KMC in Malawi. This information will build the evidence that is needed to optimise the QoL of a mother while providing KMC.

## Methods

The study is reported according to the Standards for Reporting Qualitative Research (SRQR) [16]. The SRQR checklist is included as Additional file 1: File 1.

### Study design and setting

This exploratory qualitative study employing a phenomenological approach to explore the perspectives and experiences of mothers’ QoL delivering facility-based KMC is a secondary analysis of the KMC data collected during the “Integrating a neonatal healthcare package for Malawi” project [13]. The project employed qualitative research to understand factors that influence the scale-up of newborn care interventions at low-resource health facilities from the perspective of health workers and caregivers. The research is part of the Innovating for Maternal and Child Health in Africa (IMCHA) initiative, funded in partnership with the Canadian International Development Research Centre, Global Affairs Canada, and the Canadian Institutes for Health Research. Qualitative research was conducted in collaboration between Malawian (ALNM, SS) and Canadian (MWK, MV) social scientists with the support of local and international pediatric clinicians (LN, QD, EM, DG, KK). Our project was conducted in southern Malawi, at a large tertiary-level central hospital and three secondary-level district hospitals serving as regional referral centres for their rural districts. This included two government district hospitals and one private not-for-profit mission hospital, though all hospitals provided maternal and child healthcare free of charge to patients. All four hospitals had a separate room dedicated to KMC admissions, close to nursery units.

### Study participants and recruitment

The sample was purposively drawn to include health workers and caregivers engaged in neonatal care at the four hospitals. Research staff employed by the project without a previous relationship with participants approached health workers in person or by phone and asked for an interview after briefing them on the study. Nurses, clinicians, registrars, and pediatricians working in the newborn care units were recruited at the tertiary hospital. From district hospitals, nurses, clinicians as well as district health officers, district medical officers, and district nursing officers who supervised health services delivery in their district were included. All health workers that were approached accepted participation in the study. We approached mothers, fathers, and relatives in the KMC wards during their stay at the hospital, with the support of on-site nursing officers. As reported in the primary paper [13], we enrolled caregivers of infants who had spent at least one night in the KMC ward to ensure that they have had an experience of KMC. We assumed that those that had been in the KMC ward for less than 5 h would have inadequate experience. Grandmothers, aunts, and fathers were approached with assistance from the mother. Notably, fathers are rarely at the hospital and were mostly approached during the visiting hours and were recruited irrespective of their wife’s participation in the study or not. Only two women refused to take part and cited time constraints due to the care of their babies as a limitation. A sample size of 5–10 health workers and 2–5 caregivers per site was estimated to reach data saturation based on the staffing and size of KMC wards (2–8 beds in each).

### Data collection and analysis

For the primary paper, in-depth interviews using a semi-structured topic guide and non-participatory observations in the KMC ward were conducted between May and August 2019 at the four hospitals. The interview guide was developed by ALMN and MWK and was reviewed by the team members to assess its adequacy (Additional file 2: File 2). Research guides were piloted at the tertiary hospital to refine and develop the coding framework for the primary analysis and also to ascertain its ability to capture the intended information within the local settings and it was deemed adequate after a review among the team members after assessing the transcripts from that interview [12, 13]. The interview guide covered the process of KMC practice to elicit participant experiences and perspectives of the intervention and the observation guide included descriptions of the room, infrastructure, process of KMC practice, interactions between medical staff and patients, and between peers. Interviews were conducted by a team of five qualitative researchers comprising four research nurses and a public health specialist that were employed under the IMCHA study [12, 13]. The data collectors underwent a three-day intensive training in qualitative research methods that included aspects of data collection, reflexivity, and probing for more information [17]. Participants in the primary study had no prior relationship with the interviewers before the interviews [18]. All interviews were face-to-face and were conducted in a private place within the facilities. To maximize the quality of our data, researchers summarized the key findings at the end of each interview as a form of member checking, we described the context where we conducted the primary research and included a clear explanation of the methods to ensure that our results are dependable [19]. Interviews were recorded with permission, translated in verbatim, and transcripts uploaded to NVivo 12 software (QSR International, Melbourne, Australia) for analysis. More information on the topic guide, the methodology of data collection, and primary analyses are reported elsewhere [12, 13].

Secondary analysis of the qualitative dataset was conducted on NVivo 12 software (QSR International, Melbourne, Australia) led by ALNM and MWK with member checking by TH and SS. The secondary analysis included views of both health care workers and mothers so that we provide a comprehensive view of the subject by reporting areas of convergence and divergence to maximize the credibility of our findings [19]. The secondary analysis was an iterative process with multiple discussions between ALNM and MWK and later the rest of the team to ensure that the data was correctly presented [18]. The World Health Organization (WHO) conceptualizes QoL as an individual’s perception of how their well-being is affected by their physical health, psychological state, level of independence, social relationships, and the local environment [14, 15]. While our study did not use the World Health Organization Quality of Life (WHOQOL) tool for assessment, we conducted a content analysis with a coding framework that reflected the six WHOQOL domains and their facets (Table 1).

**Table 1:** Secondary analysis coding framework

Domain code	Description
Physical	Issues around pain and discomfort, energy and fatigue, sexual activity, sleep and rest, and/or sensory functions
Psychological	Issues around positive feelings, thinking, learning, memory and concentration, self-esteem, bodily image, and appearance, and/or negative feelings
Level of independence	Issues around mobility, activities of daily living, dependence on medicinal or non-medicinal substances, communication capacity, and/or work capacity
Social relationships	Issues around personal relationships and practical social support, activities as provider/supporter
Environment	Issues around freedom, physical safety, and security, home environment, work satisfaction, financial resources, accessibility and quality of health and social care, opportunities for acquiring new information and skills, participation and opportunities for recreation/leisure activities, physical environment such as pollution, noise, traffic and climate and/or transport
Other	Spirituality, religion, personal beliefs, overall quality of life, and general health perceptions

### Ethical considerations

Ethical approval was obtained from the ethical review committees at the University of Malawi College of Medicine (P.08/15/1783) and the University of British Columbia (H15-01463-A003). Participants provided written informed consent and to safeguard their confidentiality, interviews were conducted in a private area at the health facility, demographics were recorded separately and names were not recorded in the interviews or notes.

## Results

### Characteristics of participants

Overall, 51 in-depth interviews were conducted. There were 24 interviews with caregivers, including 14 mothers, six fathers, three grandmothers, and one grandfather. Fifteen of the caregivers were self-employed and ran small-scale businesses, six were housewives, and 3 were farmers.

Twenty-seven health workers were interviewed, including 14 nurses, four clinical officers, seven district health management officials, and two pediatricians/registrars. Of the health care workers, we interviewed 17 had under 5 years of experience in the provision of KMC while 8 had over 5 years of experience.

### Findings on the mother’s quality of life

Health workers in our study reported that caregivers felt skin-to-skin contact between mother and infant was simple and not difficult to understand. However, KMC was considered a difficult practice. Mothers experienced multidimensional challenges to their QoL while delivering facility-based KMC, as further described below (Table 2).

**Table 2:** Summary of findings as per tenet of the WHOQOL and the sub-population

Type of client	Tenets of the WHOQOL framework				Implications
	Physical health	Level of independence	Social relationships and psychological state	Environment	
Mothers	Tiring position			Boredom	May have interrupted KMC sessions There is a need to create activities that can occupy the mother other than KMC There is a need to make allowance for other family members to assist in the provision of KMC There is a need for continuous training on how to handle a baby
	Fearful to handle a small baby				
Health care workers		Restricted Movements especially on self-care and food preparations	Stressful experience	Lack of edutainment	Interrupted KMC by the mothers and likely abscondment from the facility-based KMC Have edutainment and handicrafts in the facilities and lobby for more space for KMC Allow other family members to freely visit while safeguarding the privacy of other patients
			Isolation	Lack of handicraft	
				Inadequate space for KMC	
Fathers			Loneliness for the mother		Psychological stress on the mother Allow the male partner to visit and interact and assist the mother

### Physical health

Participants reported that KMC was tiring because of the constant positioning of the infant on the mother’s chest. With recommendations to deliver KMC continuously as much as possible, caregivers had to be always engaged and in the position to deliver KMC.

“The challenge is that the baby is always to be with me carried on my chest which is not an easy thing, it is a tiring job” **Mother at a district hospital**

Positioning the infant on her chest all of the time was described to be especially uncomfortable for sleeping. Mothers found it difficult to rest while constantly delivering KMC. A nurse from a district hospital reported that mothers resisted KMC because it compromised their ability to sleep:

“They (mothers) said this meant that we will always be busy and how am I going to sleep if I put the baby on my abdomen? I am tired; I can’t manage to always sleep on my back.” **Nurse at a district hospital**

### Level of independence

Positioning the infant on the mother’s chest was also associated with restricted movement and the ability to do activities. Mothers were told that they should only take breaks to go to the washroom. Mothers were monitored and judged on how long they were away from their babies.

“They said the child is supposed to be on KMC…you can’t remove them and also you can’t go outside. You need to stay inside here to keep the child warm…staying here all the time, in one place, we don’t go elsewhere.” **Mother at the tertiary hospital**

“They should remove the baby on KMC [to go to the toilet] but don’t take much time… When washing, they should remove the baby and wash in a hurry and put back the baby on KMC... Mothers have put their babies off kangaroo just to chat outside. For us, [we have] to call them. They love chatting; they forget their babies in blankets …It is not permitted that they should loiter anywhere… They are not allowed.” **Nurse at a tertiary hospital**

Mobility restrictions presented challenges to mothers’ daily living activities, particularly around food and meal preparation as cooking areas were located outside of the KMC ward. Also, observations found that some hospitals restrict access to the KMC wards to reduce the risk of infection and protect women’s privacy. However, this meant that mothers had to meet their husbands and other relatives outside of the ward. Husbands and other family members visited to bring food, which was inadequately provided by the hospital.

“We had one facility where I was working and we had budget cuts who were aiming at cost-cutting. There was an idea that food should not be provided to mothers who have babies on KMC. The rationale was that the patient was the baby and not the mom… you know, you can’t do KMC while hungry” **Pediatric clinician at the tertiary hospital**

### Social relationships and psychological state

Facility-based KMC was frequently described as an isolating experience for mothers. In addition to bringing food and supplies, visits from husbands and other family members were important to connect about family life and other children at home. Some fathers lamented about the family separation and wished they could do more to help.

“Kangaroo intervention is not easy and it’s tiring… to the mother…because the baby is always on the chest. Had it been that it is at home, we can be assisting her… She has been taking care of the child here [at the hospital] all alone…” **Father at the tertiary hospital**

Some mothers were worried that they were not used to remaining still without turning during their sleep. Their fears of accidentally hurting their infant were associated with emotional distress and difficulties sleeping.

“I understood it (KMC) well, but I was scared while practicing it…. I was scared to put a baby in a KMC position while I am sleeping. I was thinking about how am I going to be turning while asleep? And since we were told not to be turning while sleeping, I was so worried….” **Mother at a district hospital**

Overall, poor self-esteem and negative feelings were rarely discussed on their own within our study but reported in association with social isolation. For example, a pediatric clinician shared that KMC mothers may feel lonely and a nurse from a district hospital described how the isolation of KMC mothers in a separate ward was associated with feelings of low self-esteem.

“We had a very young mother who would not be keen on KMC… She didn’t want to stay in the ward by herself while all the other mothers have gone out.” **Pediatric clinician at the tertiary hospital**

“They feel isolated. [With] how our kangaroo [care] is designed, they feel isolated from the rest of the mothers and they feel they are not important enough” **Nurse at a district hospital**

### Environment

Caregiver experiences during facility-based KMC were also characterized by boredom. There was a lack of activities and entertainment for mothers. Nurses described that mothers sometimes complained that they felt like they were in a prison inside the KMC ward.

“There is a need to make it more interesting for our mothers … Long time ago in other hospitals, people were being given knitting kits… If there was a chance of a radio or even television in the units where the mothers are doing kangaroo mother care, it can be good because when the mothers see that, they wouldn’t feel like they are in prison.” **District hospital nurse**

“The mothers who have to be here all day, every day…There is no chance for entertainment, no lounge. Maybe it would be easier if there was a TV screen here…I think it’s just lying here all day, nothing else to do, not having enough food.” **District hospital nurse**

“I wash the baby’s nappies then I do nothing other than sitting and sleeping” **Mother at a district hospital**

While caregiver challenges were reported at both the tertiary hospital and district hospitals, difficulties appeared to be magnified at the secondary-level facilities where space for KMC and staffing support was more limited. While the KMC ward at the tertiary hospital was large, space for KMC was small in two out of three district hospitals. In one district hospital, the room could only fit two beds against each other without space in between, which local health officials described as inadequate and compromised the comfort of mothers practicing KMC.

“Ideally, you are supposed to have a very big room with beds for mothers doing KMC but our hospital….we have challenges with the KMC room. We just accommodate two people…I think the issue is to make sure the environment is good for the mother and baby…” **District medical officer**

## Discussion

The purpose of this study was to understand the impact of facilitating facility-based KMC on mothers’ QoL at hospitals in Malawi. While caregivers at Malawian hospitals understood KMC as a simple intervention, it was described as an exhausting experience for mothers in this study. Restricted movement, compromised sleep, discomfort due to being in one position for prolonged periods, boredom, and isolation from family members during their stay at the hospital contributed to decreased quality of life. Isolation from family members concentrated the burden of care on mothers and compromised capacity to meet daily living needs, including cooking meals and food provisioning.

Other studies on the scale-up of KMC have previously described challenges, including that mothers may get tired of being in one position for prolonged periods [7, 9, 20] resulting in sleep deprivation for the mother [21, 22]. However, to the best of our knowledge, this is the first study investigating mothers’ QoL while delivering KMC in a resource-limited setting. While studies have shown that KMC may benefit mothers’ mood and lower maternal postpartum depression (PPD) rates through increased bonding between mothers and infants [23, 24], our study suggests that compromised QoL may negatively impact KMC mothers’ wellbeing.

Our research builds on previous research reporting higher rates of maternal PPD and lowers maternal postpartum QoL among mothers of preterm infants [25–27], particularly those with infants admitted to intensive care [28–30]. Factors related to heightened caregiver anxiety and stress include the closed environment of neonatal wards that restricted access for family members, severely disturbed sleep, and poor parental knowledge concern about infant prognosis as well as costs related to hospitalization and poor family economic situation [27, 30, 31]. Boredom and lack of activities for mothers were also associated with less sleep at night, increased daytime napping, and increased rates of maternal PDD [32]. These factors were also evident for mothers while delivering KMC in a resource-limited setting in our study.

Additionally, the form of KMC practiced may have implications. While intermittent KMC was associated with improved maternal mental health [33], continuous KMC was exhausting and stressful for caregivers to undertake the care of vulnerable low birthweight infants full-time [21, 22]. KMC wards at our study hospitals in Malawi were not equipped with incubators. Because infants would be wrapped in blankets when skin-to-skin contact was not being practiced, mothers were urged to practice KMC as continuously as possible. The emphasis on continuous KMC without adequate social or hospital support reflects poor consideration for the personal toll of facilitating KMC.

Previous research highlighted that hospital layout separating the KMC ward may be associated with systematic neglect as KMC infants are considered largely in stable health and health workers prioritize other urgent care areas [12]. The current study further reveals that separation of the KMC ward may also contribute to poor maternal QoL within the Malawian context. Having a preterm infant is traditionally stigmatized in Malawi and families were ashamed of their small infants, previously hiding them within their homes after delivery [13]. The isolation of the KMC ward as its unit where KMC mothers were expected to stay until their infants grew to an adequate size may mimic traditional values and practices, leading to feelings of shame and inferiority among KMC mothers.

Healthcare workers' supportive care and appropriate KMC unit infrastructure and equipment have been shown as valuable to caregivers during KMC practice [24]. Supportive nurse mothers' interaction and staff assistance in infant care, such as expressed breastfeeding during the night, are needed [21, 22]. Allowing other caregivers within a woman’s support system, including husbands, in providing KMC supports mothers and reduces their isolation, which can also alleviate the sense of being restricted in their movements, boredom, and anxiety. Better integration of the KMC ward with the rest of the newborn and maternity care is also required.

Limitations of the study include that it is a secondary analysis of data thus our findings have to be taken cautiously and while qualitative analyses reflected the domains of the validated WHOQOL tool, our study did not use the assessment tool for data collection. The psychological and spiritual domains did not feature strongly in our results, but because we did not include explicit questions regarding the specific QoL domains, it remains unclear whether these issues were less prominent or perhaps not reported. Although conducting the study within a hospital setting could have influenced the type of responses that our participants gave, research assistants encouraged the participants to give honest answers and that what they said would not affect the receipt of services at the facilities. Our findings could also be limited by the limitations that were expressed in the primary study [12, 13]. Additionally, study findings may be limited by the specificity of a resource-limited health setting, which may limit the transferability of results to KMC units in high-income settings. Our findings may be transferable to settings that are similar to the ones we used in this study with caution that this is a secondary analysis. Cognizant that our sample size and findings may be limited, more research is needed to confirm findings from our exploratory study in other contexts. Future research should focus on assessing the quality of care of caregivers whose children are under KMC with a focus on both the mother and her extended family while using the appropriate quality of care tools in the study.

## Conclusion

Our study highlights the heavy burden placed on mothers who become the key person responsible for care during KMC and the impact it has on their daily life while hospitalized. To better support mothers during facility-based KMC, health system strengthening activities should focus on adequate staffing with appropriate training, shifting workplace culture to supporting caregiving tasks and infrastructures like cooking areas, entertainment, visitor areas to facility meetings with family members during KMC, reclining beds, and provisioning food. More focus is needed on mothers as a person with feelings, concerns, and needs and engaging their support network.

## Supplementary Information

**Additional file 1.** The SRQR checklist.

**Additional file 2.** The interview guide was developed by ALMN and MWK and was reviewed 129 by the team members to assess its adequacy.

## Data Availability

The datasets generated and/or analyzed during the current study are not publicly available due to participant privacy but are available from the corresponding author on reasonable request.
