# Supplementary material for: Mothers’ quality of life delivering kangaroo mother care at Malawian hospitals: a qualitative study
Source: Health Qual Life Outcomes. 2021 Jul 28;19:186. doi: 10.1186/s12955-021-01823-8 (PMC8317316; doi:10.1186/s12955-021-01823-8)
Supplement: Supplementary file 2 — Additional file 2. The interview guide was developed by ALMN and MWK and was reviewed 129 by the team members to assess its adequacy. [file 12955_2021_1823_MOESM2_ESM.docx]

# Healthcare workers experiences with interventions to improve neonatal health

## Topic Guide

- *Introduce yourself and ask the participant how they are doing today, etc.*
- *Introduce the project and go through the consent form with the participant*
- *Get the participant to fill out the demographics form*

| **Topic** | **Question** | **If not already brought up by participant, probe:** |
| --- | --- | --- |
| **Introduction** | 1. How long have you worked in the unit? |  |
|  | 1. Which units do you prefer to work in? |  |
|  | 1. Please describe a typical day in this ward for you | - What do you do? - What are your responsibilities? |
| **Training** | 1. First of all, can you tell me more about when you started using *_(intervention) ?* |  |
|  | 1. Can you tell me how you were trained? | - Formal or informal? - Length of training? - Who were the trainers? |
|  | 1. In your training, what did you find most helpful/useful for using ______________in your workplace? | - What did you feel was important and not important to be incorporate in training? |
|  | 1. What other things would you like to learn to better be able to use ______________ in your workplace? |  |
|  | 1. What stood out for you while learning about ______________ such as anything you found surprising or interesting? |  |
| **Initiation** | 1. I have a couple general questions about using ______________ for newborn babies at your workplace. Would you describe to me how ______________ is started at your workplace? | - Who has the authority to start? - Who actually starts it? - When is it expected to be started after authorized? - Any differences between shifts (day/night/weekend/holidays)? |
|  | 1. Would you describe factors that make it easier to start ______________? Can you provide an example? |  |
|  | 1. Would you describe factors that make it more difficult to start ______________? Can you provide an example? |  |
|  | 1. What do you do when you face challenges? Can you provide an example? | - Sources of support? - Shift differences? |
|  | 1. Would you describe any disagreements with initiating______________ that you may have observed or heard about? | - Who disagreed with who? - Why did they disagree? - What happened? - How was it resolved? - Any other examples? |
|  | 1. Can you describe a time at your workplace when there was a delay in starting ______________? | - How long was the delay? - Why do you think this happened? - What resulted in your example? |
|  | 1. Can you describe a time at your workplace when ______________ was not given? | - Why? - What happened? |
| **Monitoring** | 1. Would you describe how monitoring happens at your workplace? | - Who monitors? - When and how often? - What is monitored and why? - Where do they monitor? - Ideal vs reality? - Shift differences? |
|  | 1. Would you describe factors that make it easier to monitor ______________? Can you provide an example? |  |
|  | 1. Would you describe factors that make it more difficult to monitor ______________? Can you provide an example? |  |
|  | 1. What do you do when you face challenges? Can you provide an example? | - Sources of support? - Shift differences? |
|  | 1. Would you describe any disagreements with monitoring ______________ that you may have observed or heard about? | - Who disagreed with who? - Why disagree? - What happened? - How was it resolved? - Any other examples? |
|  | 1. Can you describe a time at your workplace when there was a delay in monitoring? | - How long? - Why? - What happened? |
|  | 1. Can you describe a time at your workplace when monitoring did not happen? | - Why? - What happened? - How often are clients not monitored? |
| **Perceptions of health care professionals** | 1. Would you describe how _*(condition)*_ would have been managed in his workplace before _*(intervention)* ? |  |
|  | 1. What has changed since ______________ was introduced? | - For district hospitals, how has this intervention affected referral rates to Queens? |
|  | 1. In your experience, can you describe situations where ______________helped the baby get well? | - What happened? - Why they think it happened? - What could be learned from this situation? |
|  | 1. In your experience, can you describe situations where ______________caused harm to the baby? | - What happened? - Why they think it happened? - What could be learned from this situation? - Did it change your opinion on ______________? |
|  | 1. Overall, what do you think about ______________? | - How does using ______________ make you feel? - What did you like about using it and what did you not like? - How has it influenced your practice? |
| **Perceptions of parents** | 1. What are some of the perceptions that mothers/guardians have of ______________? | - What are cultural beliefs around components of the intervention (i.e. depending on intervention: breastmilk, colostrum, always holding newborn, blue lights, warming boxes, etc.)? |
|  | 1. Why do you think they think this way? | - Any concerns regarding HIV positive status for those applicable? |
|  | 1. How do you explain ______________ to the mother/guardian? | - Who talks to guardians? - When/at what point and why? - Give an example of what you would say - How would the guardian receive the information you provide? - If no one talks to the parents then why not? |
|  | 1. Any examples of parents who refused? Who, why and what was done? |  |
|  | 1. Any examples of parents who initially refused and then accepted? What changed their mind? |  |
|  | 1. Any examples of parents who accepted right away? Why do you think this was? |  |
|  | 1. Would you describe how parents care for their baby while on _____________? | - Holding, changing, feeding, - Challenges - How to support, peer support |
|  | 1. What factors do you think make it easier for parents to be comfortable with ______________? Can you provide an example? |  |
|  | 1. What factors do you think makes it more difficult for parents to use ______________? Can you provide an example? | - For HIV positive parents, are there any specific challenges or concerns that parents have? |
| **Closing** | 1. Thank you. These are all the questions I had for you. Is there anything you would like us to know about your experience with ______________ or how health care workers could be supported to continue to provide ______________ to newborns? |  |

# Caretaker experiences with interventions to improve neonatal health

## Topic Guide

- *Introduce yourself and ask the participant how they are doing today, etc.*
- *Introduce the project and go through the consent form with the participant*
- *Get the participant to fill out the demographics form*

| **Topic** | **Question** | **If not already brought up by participant, probe:** |
| --- | --- | --- |
| **Introduction** | 1. What is your relationship to the newborn? |  |
|  | 1. Would you be able to tell us a little of yourself and where you are from? | - Where are you from? - What is your/your husband’s profession? - How many children do you have? |
|  | 1. How did you come to the hospital today? | - What means of transport? - How long did it take? - Were you referred here by another facility? What was the process of referrals? |
|  | 1. Please describe if you experienced any challenges coming to the hospital today | - Who is looking after your other children at home? - Cost of transport? Availability of transport? - Concerns regarding time away from home and/or job? |
|  | 1. What would make it easier for you to come to the hospital today? |  |
| **Initiation and monitoring** | 1. Would you describe how medical staff started ­­­­(the intervention) with you? | - Was consent obtained? Was it obtained before or after starting the newborn on the intervention? How did that make the you (parent) feel? - Were there any delays in initiating the intervention? If so, why did they think there was a delay? |
|  | 1. Would you describe how the medical staff explained ­­­­­­­­­­______________ to you? | - Was it sufficient? What did you want to know more? - How did the information make you feel? |
|  | 1. Do you feel the staff were sufficiently trained to start and monitor the baby on ______________? | - Why or why not? - How did that make you feel? |
|  | 1. Would you describe how medical staff looked after (monitored) the baby while on _______________? | - How often did medical staff check in? - Did you feel that was sufficient? |
|  | 1. What roles and responsibilities did parents have in monitoring/looking after their baby while on _____________? | - How did medical staff support parents in their roles? - What made doing these activities difficult? |
| **Perceptions of parents** | 1. When you first heard of ________________, what did you think of it? | - What are your understanding of the intervention? - What is said about the intervention among people in your family (i.e. husband, mothers-in-law, fathers-in-law… etc)? - What is said about the intervention among people in your community and/or religious leaders? - What are cultural beliefs around components of the intervention (i.e. breastmilk for lactation support, blue lights for phototherapy, the boxes for infant warmers, always holding the baby for KMC, etc.) |
|  | 1. When you first heard doing ­­­­­­­­­­________________ with your baby, how did that make you feel? | - Any fears? Any feelings of hope? Joy? Etc. - For especially lactation support: if HIV positive, did you have any specific concerns? |
|  | 1. Now that you have used _______________, how do you feel about it now? | - Any changes in their opinions? What changes? How did it change? Why the change in opinion? |
|  | 1. What factors do you think make it easier for you to be comfortable with ______________? Can you provide an example? | - What support can medical staff provide? |
|  | 1. What factors do you think makes it more difficult for you to use ______________? Can you provide an example? | - What challenges do you face while using the intervention? |
| **Closing** | 1. Thank you. These are all the questions I had for you. Is there anything you would like us to know about your experience with ______________ or how you could be supported to use______________ with your newborn? |  |
